# Social support as a mediator between life events and non-suicidal self-injury: evidence for urban-rural moderation in medical students

**DOI:** 10.3389/fpsyt.2025.1522889

**Published:** 2025-03-04

**Authors:** Zhumei Lin, Yiwen Zhang, Siru Kong, Qianan Ruan, Li-Li Zhu, Cheng-Han Li

**Affiliations:** ^1^ Department of Psychology, Xiamen Medical College, Xiamen, China; ^2^ Department of Neurology, The Second Affiliated Hospital of Xiamen Medical College, Xiamen, China; ^3^ Career Education Research Center, Wenzhou University, Wenzhou, China; ^4^ Emergency Department, Third Afliated Hospital of Wenzhou Medical University, Wenzhou, China

**Keywords:** non-suicidal self-injury (NSSI), medical students, social support, urban-rural differences, moderated mediation

## Abstract

**Background:**

Non-suicidal self-injury (NSSI) among medical students represents a critical public health concern, yet the protective mechanisms and their effectiveness across different demographic contexts remain poorly understood. This study investigates how social support mediates the relationship between negative life events and NSSI, while examining the moderating effect of urban-rural origins.

**Methods:**

A cluster sampling approach was employed to survey 1,130 first-year medical students (471 males, 659 females; mean age 18.15 ± 0.63 years; response rate: 98.5%) at Xiamen Medical College, including 473 urban and 657 rural students. Participants completed the Adolescent Self-Rating Life Events Checklist, Social Support Rating Scale, and Adolescent Self-Injury Questionnaire. Harman’s single-factor test confirmed no significant common method bias. Hayes’ PROCESS macro (Model 7) was used to test the moderated mediation model, with bootstrapping (5,000 resamples) for significance testing.

**Results:**

Correlation analysis revealed significant associations between life events and NSSI (*r* = 0.28, *p* <.01), life events and social support (*r* = -0.36, *p* <.01), and social support and NSSI (*r* = -0.19, *p* <.01). The mediation analysis showed that social support partially mediated the relationship between negative life events and NSSI (indirect effect = 0.01, 95% *CI* [0.002, 0.016]), accounting for 14.7% of the total effect. Life events significantly predicted social support (*β* = 0.56, *p* <.001) and NSSI (*β* = -0.02, *p* <.001). The moderation analysis revealed that birthplace significantly moderated the relationship between life events and social support (*β* = 0.16, *p* <.05), with urban students showing greater vulnerability to reduced social support (*β* = -0.14, *p* <.001) compared to rural students (*β* = -0.10, *p* <.05).

**Conclusions:**

This study reveals that while social support serves as a crucial buffer against NSSI, its protective effect varies significantly between urban and rural students. Contrary to traditional resource-based assumptions, urban students demonstrated greater vulnerability to stress-induced reduction in social support. These findings suggest the need for targeted interventions that consider students’ geographical backgrounds when developing support systems in medical education.

## 1 Introduction

Non-suicidal self-injury (NSSI) is defined as the deliberate, direct destruction of body tissue without conscious suicidal intent (1). Common forms include cutting, burning, scratching, and hitting oneself (2). Global prevalence rates among adolescents range from 17% to 37% (3, 4), with a higher rate among medical students than typical rate of NSSI (5). NSSI serves multiple functions, including emotion regulation (6), self-punishment (7), and interpersonal influence (8). While distinct from suicide attempts, NSSI is a significant risk factor for future suicidal behavior, making it a crucial target for prevention and intervention efforts (6, 9).

The transition to college life, especially for first-year medical students, introduces unique stressors such as academic pressure and adaptation challenges, potentially heightening vulnerability to NSSI (10). Medical students often face intense academic pressure, high expectations, and exposure to emotionally challenging situations, which can increase their vulnerability to psychological distress and maladaptive coping mechanisms (11, 12). Understanding the interplay of factors contributing to self-injury in this population is essential for promoting mental health and well-being.

### 1.1 Life events and NSSI

Negative life events are significant stressors that can profoundly affect adolescents’ psychological well-being (13). These events encompass a range of experiences, including academic pressure, interpersonal conflicts, family disturbances, and health-related issues (14). The Adolescent Self-Rating Life Events Checklist (ASLEC) has emerged as a widely used instrument for assessing the frequency and impact of such events on young individuals (15).

Research has consistently demonstrated a positive correlation between negative life events and NSSI among adolescents (16, 17). According to the Stress Erosion Model, negative life events can lead to heightened emotional distress, feelings of hopelessness, and a lack of perceived control, which may prompt adolescents to engage in self-injury as a maladaptive coping mechanism (18). For instance, a study by Maria et al. (19) found that adolescents who experienced a higher number of negative life events reported increased instances of NSSI. Similarly, Claes et al. (20) observed that academic stress and interpersonal problems were significant predictors of self-injury behaviors among high school students.

### 1.2 Social support and NSSI

Social support refers to the perception or experience of being cared for, valued, and part of a supportive social network (21). It encompasses emotional support, informational support, and tangible assistance received from family, friends, and significant others (22). The Social Support Rating Scale has proven to be an effective tool for measuring the extent and quality of social support among college students (23).

Extensive research has highlighted the protective role of social support in mitigating the adverse effects of negative life events on mental health outcomes (24, 25). Social support can enhance an individual’s ability to cope with stress by providing emotional comfort, practical help, and advice, thereby reducing the need to resort to maladaptive coping strategies like NSSI (26). For example, Park and Crocker (27) found that perceived social support negatively correlated with self-injury behaviors among adolescents, suggesting that higher levels of support reduce the likelihood of NSSI.

Several studies have investigated the mediating effect of social support in the relationship between negative life events and NSSI (28). Xin et al. (29) reported that social support partially mediated this relationship among Chinese adolescents, indicating that negative life events decreased perceived social support, which in turn increased the risk of NSSI. Similarly, Mogens et al. (30) found that social support served as a mediator between stressful life events and self-injury, highlighting its critical role in adolescent mental health.

The buffering hypothesis suggests that social support can cushion individuals against the harmful effects of stressors by altering their appraisal of stress and enhancing coping resources (31). In the context of NSSI, social support may reduce feelings of isolation and hopelessness, provide alternative coping strategies, and increase resilience (32). This mediating role underscores the importance of fostering supportive environments for adolescents to prevent self-injury behaviors.

### 1.3 Urban-rural differences

The role of geographical background in shaping mental health outcomes has gained increasing attention in recent research. Urban and rural environments present distinct social, cultural, and economic contexts that may influence how individuals experience and respond to stress (33). Rural communities often feature stronger social bonds and traditional support networks, potentially offering protective factors against psychological distress (34). Conversely, urban environments, while typically providing better access to mental health resources, may foster greater social isolation and competitive pressure (35).

Studies examining urban-rural differences in mental health outcomes have produced mixed results. While some research highlights the disadvantages faced by rural adolescents due to limited resources and access to support services (36, 37), others suggest that urban youth may experience unique stressors related to social fragmentation and heightened academic competition (38). However, the specific moderating effect of urban-rural background on the relationship between life events, social support, and NSSI remains understudied.

### 1.4 Research gaps

Despite substantial research exploring the relationships among negative life events, social support, and self-injury behavior in adolescents, several critical gaps remain. Firstly, while the mediating role of social support has been examined in general adolescent populations (27, 29), there is a lack of studies focusing on specific subgroups such as first-year medical students who may experience unique stressors due to the demanding nature of medical education (10). Medical students often face intense academic pressure, high expectations, and exposure to emotionally challenging situations (11, 12).

Secondly, the moderating effect of birthplace (urban vs. rural) on the relationship between negative life events and self-injury behavior, particularly through the mediating role of social support, is under-researched. While some studies have considered the influence of urban-rural differences on adolescent mental health (33, 38), few have integrated this variable into a comprehensive moderated mediation model.

### 1.5 Theoretical framework and hypotheses

While the stress-buffering hypothesis traditionally conceptualizes social support as a moderator of stress effects, our study adopts a mediation approach based on emerging evidence that negative life events can actively erode social support resources, particularly during critical developmental transitions (28). This perspective aligns with the stress erosion model (39), which suggests that stressors can systematically diminish social support over time. In the context of first-year medical students, who are experiencing significant life transitions, social support networks are particularly vulnerable to disruption (10). Therefore, examining social support as a mediating mechanism rather than a stable moderating resource better captures the dynamic nature of stress-support relationships during this critical period.

**Figure 1:**
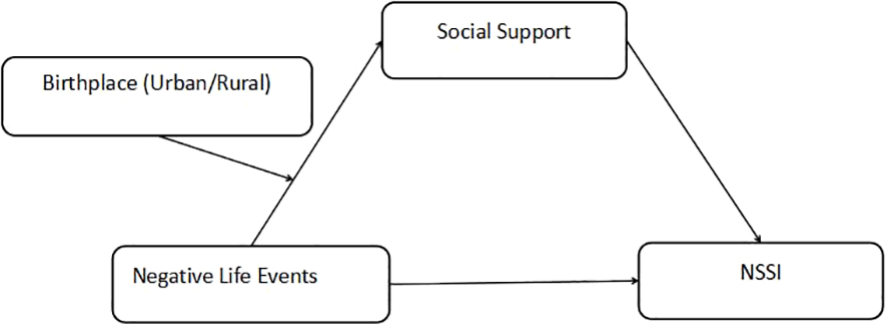
Theoretical hypothesis model.

We propose an integrated theoretical framework to understand the complex pathways leading to NSSI among medical students (**Figure 1**). This framework posits that negative life events trigger a cascade of psychological processes: they first impact individuals’ cognitive appraisal and emotional regulation capacities, potentially eroding their perceived social support, which in turn may lead to maladaptive coping mechanisms such as NSSI (18). Moreover, this process likely operates differently across various socio-ecological contexts, particularly between urban and rural environments, due to distinct social network structures and cultural values (33). This theoretical integration not only helps explain the direct impact of stressors on self-injury but also illuminates the mediating role of social support and the moderating influence of urban-rural birthplace.

Based on our theoretical framework and existing literature, we propose three hypotheses:

1. Negative life events will positively predict NSSI among first-year medical students;

2. Social support will mediate the relationship between negative life events and NSSI, with negative life events reducing perceived social support, which in turn increases NSSI risk;

3. The indirect effect of negative life events on NSSI through social support will be moderated by urban-rural background, with differences in social network structures and cultural values affecting the strength of this relationship.

## 2 Method

### 2.1 Participants and procedures

A cluster sampling method was employed, where first-year medical students at Xiamen Medical College were surveyed by class using an electronic questionnaire. Data collection was conducted during regularly scheduled class periods after obtaining approval from course instructors. Students were informed about the study one week prior through their class representatives. On the day of data collection, trained research assistants explained the study purpose, voluntary nature of participation, and confidentiality procedures. Students who chose not to participate were free to engage in quiet personal activities during the data collection period.

A total of 1,147 questionnaires were collected, with 17 invalid questionnaires excluded, resulting in 1,130 valid responses (98.5% validity rate). The questionnaires can only be submitted when all the items were completed. There were no blank answers and the criteria for “invalid” responses was a unified answer. The average age of the participants was 18.15 ± 0.63 years, including 471males and 659 females. Among them, 473 students were from urban areas and 657 were from rural areas. The study received approval from the Medical Ethics Committee of Xiamen Medical College (No. 20220901016), and participants provided informed consent, being assured that their data would be used exclusively for research purposes and kept confidential.

### 2.2 Measures

#### 2.2.1 Adolescent self-rating life events checklist

The ASLEC was used to assess whether participants had experienced corresponding negative life events in the past 12 months and the severity of their impact. The scale consists of 27 items across six dimensions: interpersonal relationships, academic pressure, punishment, loss, health adaptation, and others. Each item is scored on a 5-point scale, ranging from 0 (no occurrence, no impact) to 4 (occurrence with extreme impact). A higher total score indicates greater exposure to negative life events. The Cronbach’s *α* coefficient for this scale was 0.96.

#### 2.2.2 Social support rating scale

The Social Support Rating Scale for College Students, developed by Ye Yuemei et al. (23), was used to measure social support. This scale consists of 17 items across three dimensions: objective support, subjective support, and support utilization. Each item is rated on a 5-point scale, from 1 (not applicable) to 5 (fully applicable), with a higher total score indicating greater social support. The Cronbach’s *α* coefficient for this scale was 0.91.

The Social Support Rating Scale’s multidimensional structure allows us to capture the complex nature of students’ support networks during their transition to medical school. The objective support dimension measures both pre-existing family support and newly formed institutional support. The subjective support dimension assesses students’ perceived support availability across different contexts, while the support utilization dimension captures their ability to mobilize both existing and new support resources.

#### 2.2.3 Adolescent self-injury questionnaire

This 19-item questionnaire, developed by Feng Yu (40), was used to measure self-injury behaviors in the past 12 months. The frequency of self-injury and the degree of physical harm caused were multiplied to evaluate the behavior. The frequency was divided into four levels: 0 times, 1 time, 2–4 times, and 5 or more times, and the degree of harm was rated on five levels: none, mild, moderate, severe, and extremely severe. The total score was calculated by summing all items, with a higher score indicating more severe self-injury. The Cronbach’s *α* coefficient for this scale was 0.85.

All measures used in this study are well-validated instruments with established psychometric properties in Chinese populations. The high internal consistency coefficients obtained in our sample (*α* = 0.85-0.96) align with previous validation studies. The factor structures of these measures have been repeatedly confirmed in similar populations, supporting their construct validity for our study context.

### 2.3 Data analysis

SPSS 26.0 was used for descriptive and correlational analyses, and the PROCESS macro (version 3.5) developed by Hayes was employed to examine the mediation and moderation effects. PROCESS Model 7 was selected because it specifically tests our theoretical framework of moderated mediation, where the relationship between the predictor (life events) and mediator (social support) is moderated by a third variable (urban-rural background). This model aligns with our hypothesis that the strength of the mediation effect varies across different geographical contexts. Alternative models (e.g., Models 8, 14, or 15) were considered but rejected as they did not match our theoretical proposition that geographical background primarily influences how individuals mobilize and maintain social support when facing negative life events, rather than moderating other pathways in the model.

While our data showed significant correlations between gender and other variables, we chose birthplace as our primary moderator for several reasons:

1. Theoretical basis: Our research aimed to extend previous findings on urban-rural differences in mental health outcomes and access to support resources, addressing a key gap in the literature regarding how geographical background influences the relationship between life events and NSSI.
2. Novelty: While gender differences in NSSI have been extensively studied (41), the moderating role of birthplace remains understudied, particularly in the context of medical education.
3. Practical implications: Understanding urban-rural differences has important implications for developing targeted interventions that consider geographical disparities in mental health resources and support systems.

## 3 Results

### 3.1 Variance inflation factors and common method bias test

Prior to main analyses, we conducted multicollinearity diagnostics. Variance Inflation Factors for all predictors were well below the conventional threshold of 10 and tolerance values were all above 0.20. Since the data were collected via self-report, there may be concerns about common method bias. Harman’s single-factor test was conducted, revealing that 17 factors had eigenvalues greater than 1, with the first factor explaining 18.1% of the variance, which is below the critical threshold of 40%. Therefore, common method bias was not a significant issue in this study.

### 3.2 Descriptive statistics and correlation analysis

The descriptive statistics and correlation analysis of the variables are presented in **Table 1**. There was a significant negative correlation between adolescent life events and social support, and a significant positive correlation between life events and self-injury behavior. Social support was significantly negatively correlated with self-injury behavior. While the zero-order correlation showed a negative relationship (*r* = -0.19, *p* <.001), the regression coefficient in the mediation model showed a positive relationship (*β* = 0.05, *p* <.05) when controlling for life events. This sign reversal represents a suppression effect, where the shared variance between life events and social support reveals the complex nature of these relationships. The positive coefficient in the mediation model represents the unique effect of social support on NSSI after accounting for the strong negative relationship between life events and social support (*r* = -0.32).

**Table 1:** Descriptive statistics and correlation analysis results (N=1130).

Variable	Mean ± SD	1	2	3	4	5	6
1. Gender	–						
2. Birthplace	–	-0.05					
3. Age	18.15 ± 0.63	-0.02	-0.03				
4. Life Events	39.79 ± 10.60	-0.06	0.02	0.02			
5. Social Support	66.90 ± 13.57	-0.15^**^	-0.02	0.02	-0.36^**^		
6. NSSI	0.35 ± 2.09	-0.01	-0.00	0.02	0.28^**^	-0.19^**^	–

Gender and birthplace were coded as dummy variables (Male, 0; Female, 1; Urban, 0; Rural, 1). ^**^ *p* <.01. The same as follows.

### 3.3 Moderated mediation effect test

**Figure 2:**
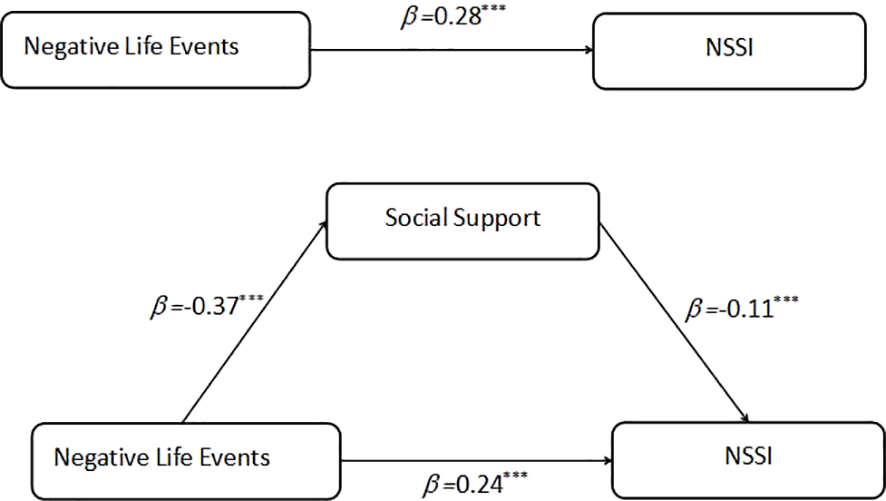
Mediator model depicting direct and indirect effects of life events on NSSI. ***p < .001.

First, the mediation effect of social support between adolescent life events and self-injury behavior was tested (**Figure 2**). Using Hayes’ PROCESS macro (Model 4), the results showed that, after controlling for gender and birthplace, life events significantly positively predicted self-injury behavior (*β* = 0.28*, p* <.001). After introducing the mediating variable, social support, life events still significantly positively predicted self-injury behavior (*β* = 0.24*, p* <.001) and negatively predicted social support (*β* = -0.37*, p* <.001), while social support negatively predicted self-injury behavior (*β* = -0.11*, p* <.001). A bias-corrected bootstrap test (with 5,000 resamples) confirmed that the mediation effect of social support was significant (effect size = 0.01, 95% *CI* [0.002, 0.016]). The model explained 16% of the variance in social support (*R²* = .16) and 9% of the variance in NSSI (*R²* = .09). Effect size analyses revealed that the model had medium effects on social support (*f²* = 0.19) and small to medium effects on NSSI (*f²* = 0.099). The mediation effect accounted for 14.7% of the total effect of life events on NSSI. This indicates that life events directly influence self-injury behavior and indirectly affect self-injury behavior through social support.

Second, the moderated mediation model was tested to examine whether birthplace moderates the relationship between life events, social support, and self-injury behavior. Hayes’ PROCESS macro (Model 7) was used for the analysis (**Table 2**). The results indicated that life events significantly predicted social support (*β* = -0.56, *p* <.001), and the interaction between life events and birthplace also significantly predicted social support (*β* = 0.16, *p* <.05). Additionally, social support significantly predicted self-injury behavior (*β* = 0.05, *p* <.001), and life events significantly negatively predicted self-injury behavior (*β* = -0.02, *p* <.001).

**Table 2:** Moderated mediation model test (N=1130).

Regression Equation		*R*	*R^2^*	*F*	*coeff*	*t*
Outcome Variable	Predictor Variables					
Social Support		0.40	0.16	53.45^***^		
	Gender				-4.70	-6.24^***^
	Birthplace				-0.51	-0.68
	Life Events				-0.56	-10.35^***^
	Life Events × Birthplace				0.16	2.24^*^
NSSI		0.30	0.09	36.59^***^		
	Gender				-0.03	-0.26
	Social Support				0.05	7.87^***^
	Life Events				-0.02	-3.52^***^

*p < .05, ***p < .001.

The observed sign reversal between the zero-order correlation (*r* = -0.19) and the regression coefficient (*β* = 0.05) represents a statistical suppression effect. This occurs because life events share substantial variance with both social support and NSSI. When controlling for life events in the mediation model, the remaining unique variance between social support and NSSI reveals a positive relationship. This suppression effect suggests that the relationship between social support and NSSI is more complex than a simple negative correlation.

These findings suggest that birthplace moderates the first part of the mediation process. To further explore the moderation effect of birthplace, a bias-corrected bootstrap test with 5,000 resamples was used to examine the mediating effect of social support on the relationship between life events and self-injury behavior in participants from different birthplaces. The results are shown in **Table 3**.

**Table 3:** Moderated mediation effect by birthplace (N=1130).

Mediating Variable	Birthplace	Effect Size	Boot SE	Boot CI Lower	Boot CI Upper
Social Support	Urban	0.009	0.004	0.002	0.019
	Rural	0.007	0.003	0.001	0.014

**Figure 3:**
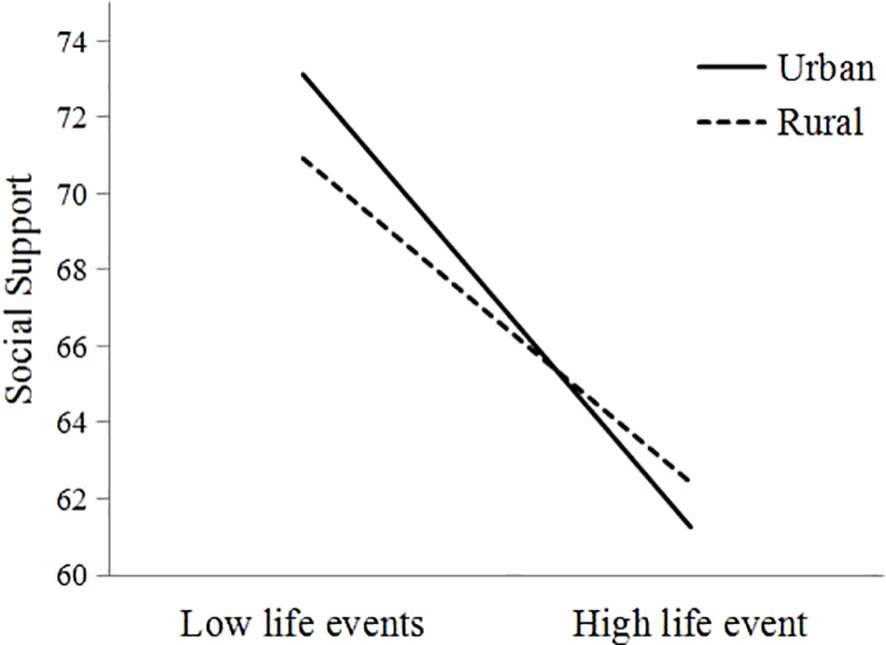
The moderating effect of birthplace on life events and social support.

Finally, a slope plot was created to explore the moderation effect of birthplace (see **Figure 3**). The negative predictive effect of life events on social support for participants from urban areas (*β* = -0.14, *p* <.001) was significantly stronger than for participants from rural areas (*β* = -0.10, *p* <.05), indicating that life events had a greater impact on the social support of urban participants.

## 4 Discussion

The present study aimed to explore the complex interplay between adolescent life events, social support, and self-injury behavior among first-year medical students. Specifically, we investigated whether social support mediates the relationship between negative life events and self-injury behavior, and examined whether birthplace (urban vs. rural) moderates this mediation effect, particularly in the relationship between negative life events and social support. Our findings revealed that social support partially mediates the relationship between negative life events and self-injury behavior, indicating that negative life events directly increase self-injury behavior and also do so indirectly by reducing social support. Additionally, we found that birthplace moderates the mediation effect; the negative impact of life events on social support was more pronounced among students from urban areas compared to those from rural areas.

Our findings should be interpreted within the context of NSSI prevalence rates across different populations. This elevated prevalence among medical students may be attributed to several factors unique to medical education, including intense academic pressure, exposure to human suffering, perfectionist tendencies, and the challenging transition to clinical training (12). The higher rates in medical students compared to their general adolescent peers underscore the importance of developing targeted interventions for this vulnerable population. Our study’s focus on first-year medical students is particularly relevant as this transitional period often coincides with peak stress levels and adaptation challenges (42). Understanding the specific pathways through which negative life events lead to NSSI in this population, particularly through the mediating role of social support, provides valuable insights for developing preventive strategies.

### 4.1 Mediation of social support

Our finding that social support partially mediates the relationship between negative life events and self-injury behavior aligns with and extends the existing literature on adolescent mental health (25, 31). Previous studies have consistently shown that negative life events are significant predictors of non-suicidal self-injury (NSSI) among adolescents (17, 18). Negative life events often lead to increased psychological distress, which can trigger maladaptive coping mechanisms such as self-injury (18). Our study corroborates these findings within the specific context of first-year medical students, a group that encounters unique stressors associated with the rigors of medical education (33).

The mediating role of social support suggests that negative life events may diminish perceived social support, which in turn increases the likelihood of self-injury behavior. Our findings revealed that negative life events significantly predicted decreased social support (β = -.32, p <.001), which warrants further discussion. This relationship can be understood through several mechanisms. First, when individuals experience negative life events, they may withdraw from social interactions due to increased stress and emotional burden, leading to reduced social support (25). This aligns with stress-generation theory (44), which suggests that stressful life events can disrupt existing social relationships and hinder the formation of new supportive connections. Second, negative life events may alter students’ perception and utilization of available social support (45). For example, academic failures or personal setbacks might lead to feelings of shame or unworthiness, making students less likely to seek help from their support network. Third, some negative life events (such as family conflicts or relocation) directly impact students’ support systems by removing or straining existing support relationships (46). For medical students specifically, the intense academic workload and time constraints following negative life events may further limit their opportunities to maintain and develop supportive relationships (12). This is consistent with the stress-buffering hypothesis, which posits that social support can mitigate the adverse effects of stress on mental health outcomes (31). By demonstrating this mediation effect, our study extends prior research that has highlighted the protective function of social support against NSSI. Specifically, it underscores the importance of social support in the well-being of first-year medical students, who may be particularly vulnerable due to academic pressures and transitional challenges (42, 43).

While the effect sizes in our mediation and moderation analyses were relatively small, these findings are consistent with previous research in psychological and educational contexts where multiple factors influence complex behavioral outcomes (47). The magnitude of these effects should be interpreted within the broader context of NSSI research, where even small effects can have practical significance given the serious nature of self-injury behaviors. Moreover, these effect sizes are comparable to those reported in similar studies examining psychosocial mediators of mental health outcomes (28, 29).

Moreover, our findings contribute to the understanding of how social support functions as a mechanism through which negative life events influence self-injury behavior. While previous studies have identified the direct effects of negative life events and social support on NSSI (28–30), our study provides empirical evidence of the indirect pathway, highlighting that enhancing social support may be an effective intervention point to reduce self-injury behaviors among medical students.

However, several important contextual factors warrant careful consideration when interpreting our findings. First, our social support measures capture a composite of both background and current support networks. The Social Support Rating Scale includes items that assess both long-standing family support (“My family supports my career choice”) and current institutional support (“I can get help from classmates when needed”). While this comprehensive approach provides a holistic view of students’ support resources, it may obscure important distinctions between different sources of support.

Second, the physical separation from traditional support networks represents a significant contextual factor, particularly for rural students who may experience greater geographical displacement when attending urban medical schools. Our findings of rural students’ greater resilience in maintaining social support levels (*β* = -0.10, *p* <.05) despite this displacement suggests that the quality and stability of support relationships may be more important than physical proximity. However, we acknowledge that our cross-sectional design limits our ability to track how support patterns evolve over time as students adjust to this separation.

Third, medical education presents unique stressors that may require specific forms of support distinct from those needed for general life stressors. These include (1)Academic pressures specific to medical training; (2) Exposure to clinical situations that may be emotionally challenging; (3) Professional socialization processes unique to medical education; (4) Competition within the medical school environment.

While our study primarily employed a mediation model, this approach complements rather than contradicts the stress-buffering hypothesis. The mediation model captures the dynamic process through which negative life events erode social support over time, while the stress-buffering hypothesis explains the protective mechanism of existing social support. In medical students specifically, this dual framework helps explain both how stressors can deteriorate support networks (mediation) and how robust social support systems can protect against the impact of stressors (buffering). This integration is particularly relevant given the unique challenges faced by medical students, where both the erosion of existing support networks and the buffering effects of maintained support play crucial roles in mental health outcomes.

The differential effectiveness of social support between urban and rural students may reflect varying abilities to adapt existing support mechanisms to these specific challenges. Urban students’ greater vulnerability to stress-induced reduction in social support (*β* = -0.14, *p* <.001) might indicate that their pre-existing support networks are less adaptable to medical school-specific stressors, despite potentially having better access to formal support resources.

### 4.2 Moderation of birthplace

In exploring the moderating effect of birthplace, we found that the negative impact of life events on social support was more pronounced among students from urban areas compared to those from rural areas. The stronger impact of negative life events on social support among urban students can be understood through several theoretical frameworks. First, the social fragmentation theory suggests that urban environments, despite their resource advantages, often feature more transient and superficial social connections (35). Second, the stress-buffering hypothesis (48) helps explain why rural students might be more resilient: their typically stronger community ties and extended family networks provide multiple layers of social protection against stressors. Third, the cultural value orientation framework suggests that rural communities in China maintain stronger collectivistic values, which may facilitate more stable social support systems even under stress (34). Urban students, conversely, often operate within more individualistic social structures that may be more vulnerable to disruption during stressful periods.

This finding is intriguing and adds a new dimension to the literature, as previous research has often focused on the disadvantages faced by rural adolescents due to limited resources and access to support services (36, 37). One possible explanation for our finding is that, facing environments with higher levels of competition and social isolation, urban students may experience a deep feeling of loneliness and desperateness despite being frequently instrumentally supported by their parents (49), leading to a greater reduction in perceived social support when confronted with negative life events. The stronger negative association between life events and social support among urban students (*β* = -0.14 vs. *β* = -0.10) can be understood through several theoretical frameworks. The social capital theory suggests that urban environments, despite their resource advantages, often feature more fragmented social networks and weaker community bonds (50). This aligns with recent research showing that urban Chinese youth experience greater social atomization and reduced family cohesion compared to their rural counterparts (Koo, 2021) (34). Additionally, the cultural-ecological model proposed by Bronfenbrenner suggests that urban students’ greater vulnerability may stem from the interaction between individualistic urban values and the collective demands of medical education, creating unique stressors that can destabilize support networks (51).

Additionally, rural students might benefit from stronger community ties and familial relationships that provide a buffer against stress (33). The collectivist culture prevalent in many rural areas may foster a sense of belonging and mutual support, which can enhance resilience in the face of adversity (34). This suggests that socio-cultural factors associated with birthplace play a significant role in shaping the availability and effectiveness of social support networks.

Our results also highlight the need to consider environmental and cultural contexts when developing interventions aimed at reducing self-injury behaviors. For urban students, interventions might focus on strengthening social networks and fostering community connections to counteract the potential isolation and competitive pressures they face (35, 52). For rural students, leveraging existing community and familial support structures may enhance the effectiveness of mental health initiatives (33).

An unexpected aspect of our findings is the directionality of the moderating effect of birthplace. Contrary to some expectations that rural students might be more adversely affected by negative life events due to fewer resources, our study suggests that urban students may be more vulnerable in terms of reduced social support leading to self-injury behaviors. This highlights the complexity of socio-environmental factors and indicates that assumptions based on resource availability may not fully capture the nuances of social support dynamic (53).

In a broader context, our study emphasizes the importance of tailored approaches in mental health interventions. Recognizing the different ways in which negative life events and social support interact across populations can inform more effective strategies to prevent NSSI (30). For first-year medical students, programs that enhance social support networks and provide coping skills for managing stressors specific to medical education may be particularly beneficial.

### 4.3 Limitations and future implications

Despite providing valuable insights, this study has several limitations. First, the cross-sectional design limits our ability to infer causality between negative life events, social support, and self-injury behavior. Second, medical students represent a distinct population and the sample comprised only first-year medical students from a single institution, which may affect the generalizability of the findings to other populations. While previous research suggests medical students may experience unique stressors, our study cannot directly compare NSSI patterns between medical and non-medical students. Third, while our study identified significant urban-rural differences, several potential confounding factors should be considered. Socioeconomic status (SES) often correlates with urban-rural residence in China, potentially influencing both access to support resources and stress responses. Additionally, pre-existing mental health conditions and previous exposure to medical environments may vary systematically between urban and rural students. Although our analysis controlled for basic demographic variables, these potential confounders might affect the interpretation of geographical background effects. Finally, the Chinese cultural context, with its distinct collectivistic values and educational pressures, may influence the manifestation of these relationships differently than in Western contexts, and social desirability bias could have influenced responses, particularly regarding sensitive topics like self-injury behaviors.

## 5 Conclusion

In summary, this study demonstrates that social support partially mediates the relationship between negative life events and self-injury behavior among first-year medical students. Negative life events not only directly increase the likelihood of self-injury but also indirectly influence it by diminishing perceived social support. Additionally, birthplace moderates this mediation effect; urban students experience a greater reduction in social support in response to negative life events compared to rural students. These findings highlight the critical role of social support in mitigating self-injury behaviors and suggest that interventions aimed at strengthening social networks—especially for urban students—may be effective in reducing such behaviors.

## Data Availability

The raw data supporting the conclusions of this article will be made available by the authors, without undue reservation.
